# Retrospective analysis of clinical characteristics and risk factors of differentiated thyroid cancer in children

**DOI:** 10.3389/fped.2022.925538

**Published:** 2022-09-12

**Authors:** Chun Chen, Lei Hang, Yan Wu, Qing Zhang, Yifei Zhang, Jun Yang, Jin Xie, Jingrong Lu

**Affiliations:** ^1^Department of Otorhinolaryngology-Head and Neck Surgery, Xinhua Hospital, Shanghai Jiao Tong University School of Medicine, Shanghai, China; ^2^Ear Institute, Shanghai Jiao Tong University School of Medicine, Shanghai, China; ^3^Shanghai Key Laboratory of Translational Medicine on Ear and Nose Diseases, Shanghai, China; ^4^Tianhua College, Shanghai Normal University, Shanghai, China

**Keywords:** differentiated thyroid cancer, recurrence, complication, children differentiated thyroid cancer, children

## Abstract

**Background:**

The incidence rate of children with thyroid cancer has an increasing trend. This study aimed to investigate the clinical characteristics and therapeutic approaches of differentiated thyroid cancer (DTC) in Chinese children.

**Materials and methods:**

From January 1998 to March 2022, 52 cases undergoing surgical resection in Xinhua Hospital affiliated to Shanghai Jiao Tong University were divided by age (≤ 7 years old: *n* = 14 and 8–13 years old, *n* = 38). Treatment methods and clinical features were analyzed to evaluate prognostic factors for oncological outcomes.

**Results:**

Among the 52 cases, the proportion of local invasion in the pre-school group was found to be higher than that in the school-age group (*p* = 0.01). T stage was significantly different between the two groups (*p* ≤ 0.05); the proportion of T_1–2_ was higher in the school-age group (32 cases, 84.2%), while the proportion of T_4_ was higher in the pre-school group (6 cases, 42.8%) relatively. The postoperative complication rate was dramatically higher in pre-school children (*p* ≤ 0.05). Additionally, the total thyroidectomy rate in the non-recurrent group was slightly higher than that in the recurrent group (*p* ≤ 0.05). Over half of the recurrent cases had low T stage and low ATA (American Thyroid Association) risk levels at initial diagnosis (78.3 and 51.4%).

**Conclusion:**

The local invasion, tumor stage, and recurrent laryngeal nerve (RLN) injury rates of the pre-school group were higher than that of the school-age group, where young age served as a potential hazard in DTC children. Hence, surgeons should emphasize high-risk features and optimize individualized surgical procedures for DTC children.

## Introduction

Thyroid cancer has become the most prevalent endocrine cancer in children, and the incidence rate of differentiated thyroid cancer (DTC) has increased rapidly in the last decade (1). DTC (papillary and follicular thyroid carcinoma) originated from follicular cells is the most common histologic subtype of thyroid cancer. Previous studies have indicated that the high incidence rate of thyroid cancers in children may have an underlying link to height and body mass index (BMI) in the process of growth (2). Meanwhile, dust and radiation exposure has been reported as a potential risk leading to the increased diagnosis of earlier-stage thyroid cancers (3).

Symptoms in children are atypical at initial diagnosis, so thyroid cancer is often diagnosed in an advanced stage or after the peripheral invasion and distant metastases. Previous studies have confirmed that the range of tumor invasion is more extensive, as is often the case, the rate of distant metastases and locoregional relapse is higher in DTC children than adults (4, 5). Fortunately, mortality after thyroidectomy in well-differentiated pediatric individuals is very low (6, 7).

Compared to adolescents, the clinical manifestation of DTC in preadolescent children is more aggressive, with a higher recurrence and distant metastases rate (5). A recent study has unveiled that lymph node metastases were found in over 40% of children with DTC (1). Since another previous study has found a higher proportion of girls than boys in children with thyroid cancer, the researchers hypothesized that the difference may be induced by estrogen, which may be a latent growth factor for malignant thyroid cells (8). On the contrary, a subsequent clinical study has pointed out that transient postoperative complications after total thyroidectomy in children were frequent relatively. Children are in the osteogenic stage of development, so the inadvertent section of parathyroid gland could lead to serious consequences and prolonged impacts on the quality of life (9). However, a number of studies were concerned with the characteristics of adolescents that in very young children are not well discussed, especially those less than 14 years old (10).

This study presents a retrospective analysis focusing on 52 DTC children who underwent thyroidectomy in our department to explore the differences in general information, clinical manifestations, TNM stages, ATA risk levels, cervical lymph node metastases, therapeutic approaches, and postoperative complications. It is important to sum up experience from limited case resources to investigate the possible factors of prevention and treatment protocols in treating young pediatric DTC patients.

## Materials and methods

### Patients and methods

From January 1998 to March 2022, the clinical data involved 52 DTC aged less than or equal to 13 years old who were treated at Xinhua Hospital Affiliated to Medical School of Shanghai Jiao Tong University by the same senior head and neck surgeon team (ethics code: XHEC-WJW-2022-272). Surgical procedures were designed by best indicator regarding tumor extent, lymph nodes invasion, and general status. According to the Chinese situation, the enrolled patients were divided into the pre-school age group (≤ 7 years old) and the school-age group (8–13 years old) regarding the individual difference, organ development, and life pattern of the two groups. The clinical characteristics of pre-school age and school-age groups were compared, and the relevant factors of recurrent and non-recurrent groups were analyzed.

Data were collected from their parents and otolaryngologists in our department, including age, gender, initial symptoms, local invasion, TNM stage, tumor size, surgical procedures, ATA (American Thyroid Association) risk levels, locoregional relapse, distant metastases, postoperative complications, and treatment approaches. All cases were primary tumors with complete clinical data and follow-up results. TNM stage was assessed based on the 8th edition of American Joint Committee on Cancer (AJCC) guidelines (11). The clinical characteristics and symptoms are defined in Table 1.

**TABLE 1:** Definitions of clinical characteristics and symptoms.

Characteristics/symptoms	Definition
Local invasion (11)	Spread outside of the thyroid capsule with invasion into cervical structures such as strap muscles, trachea, larynx, vasculature, esophagus, or recurrent laryngeal nerve.
Postoperative hemorrhage	Hemorrhage within 24 h after surgery and requires reoperation.
Transient RLN injury	Postoperative dysphagia and/or hoarseness and the symptoms are relieved within 6 months.
Permanent RLN injury	Symptoms of RLN neuropraxia persists 6 months after operation, with or without dyspnea.
Transient hypocalcemia	At least one calcium plasma level < 2.1 mmol/L with clinical symptoms of hypocalcemia like hand and foot convulsions or numbness. Symptoms disappear after calcium supplementation in 3 days after operation.
Permanent hypocalcemia	Take calcium and/or regular intramuscular injection of vitamin D more than 6 months after thyroidectomy.
ATA risk level (11)	Low risk: No local or distant metastases or all macroscopic tumor has been resected or no tumor invasion of locoregional tissues or structures or no aggressive histology or vascular invasion. Intermediate risk: Microscopic invasion into the perithyroidal soft tissues at initial surgery or cervical lymph node metastases or tumor with aggressive histology or vascular invasion. High risk: Macroscopic tumor invasion or incomplete tumor resection or distant metastases.

RLN, recurrent laryngeal nerve; ATA, American Thyroid Association.

### Microsatellite instability-PCR

Total RNA was extracted from tumor tissue and normal control paraffin sections using TRIzol reagent and reverse transcribed by the companion diagnostic sequencing kit (Takara, Tokyo, Japan). Single-nucleotide marker sites (*BAT25, BAT26, MONO-27, NR21, NR24*) were detected by Chaoxin Gene Biological Incorporation (Shanghai, China) (12). Every sample was examined three times. Tumor with two or more unstable sites was defined as MSI-H (microsatellite instability high); tumor with one unstable site was defined as MSI-L (microsatellite instability low); tumor without unstable sites was defined as MSS (microsatellite stable).

### Statistical analysis

SPSS 23.0 was used in the statistical analysis. Measure data were described as mean ± standard deviation (SD). An independent sample *T*-test was used to analyze age, tumor diameter, and follow-up time of pre-school and school-age groups along with recurrent and non-recurrent groups. Data analysis by ANOVA was required to prove the homogeneity of variance from normal distribution data. If one of the above conditions was not met, non-parametric test could be used instead. Power analysis was utilized to screen out and examine the efficiency of indicators. The χ*^2^* test was used to assess the diagnosis, TNM stage, ATA risk levels, surgical procedures, histology, postoperative complications, and other clinical conditions. The progression-free survival rate (PFS) in both age groups was evaluated by Kaplan–Meier curves and log-rank test. The significance level was set as *p* < 0.05.

## Results

In the 24-year time span, the 52 cases were divided into the pre-school group (≤ 7 years old, *n* = 14) and the school-age group (8–13 years old, *n* = 38) (Table 2). The histological subtype of all cases was papillary carcinoma. Univariate factor analysis of risk factors was examined by power analysis, the Kaiser–Meyer–Olkin (KMP) value was 0.78, and the *p-*value of the Bartlett test was significant (*p* ≤ 0.001), which indicated no significant difference in the degree of correlation between variables, and our data were suitable for factor analysis. Although no obvious difference existed in gender between the two groups, the proportion of female patients in the school-age group was slightly higher (63.2 versus 50.0%, *p* > 0.05). As shown in Table 2, there was no statistically significant difference between the two groups in the initial symptoms of foreign body sensation or cervical mass (*p* = 0.34, *p* = 0.83). However, the findings observed that pre-school children had a higher proportion of local invasions such as strap muscles, trachea, or recurrent laryngeal nerve (6 cases, 42.9%, *p* = 0.01). Regarding the tumor size, no significant difference was observed between pre-school and school-age children (*p* > 0.05). Additionally, it was found that T-stage showed a significant difference between the two groups (*p* = 0.04); the proportion of T_1–2_ stage cases was higher in the school-age group (32 cases, 84.2%). In comparison, the proportion of T_4_ stage cases was higher in the pre-school group (6 cases, 42.8%). There was no concordance in N and M stages between the two groups. According to the 8th AJCC guideline, no statistically significant difference existed regarding ATA risk levels between the two groups, while the proportion of high-risk cases in the pre-school group (5 cases, 35.8%) was higher than that in the school-age group (8 cases, 21.1%). Total thyroidectomy rate in the school-age group (32 cases, 84.2%) was found to be higher than that in the pre-school group (7 cases, 50.0%, *p* = 0.01). There was no statistical difference in lymph node dissection, hypocalcemia, and locoregional relapse rate between pre-school and school-age groups (*p* = 0.09, *p* = 0.95, *p* = 0.74). However, the pre-school patients showed statistically significance in increased transient RLN injury incidence rate versus school-age patients (21.4% versus 0, *p* = 0.02). No cases suffered from postoperative hemorrhage, permanent hypocalcemia, or permanent RLN paralysis.

**TABLE 2:** Analysis of the clinical data of DTC in pre-school and school-age children.

Characteristic	Pre-school group	School-age group	X^2^(t)	*p-*value	Total
	*n* = 14	*n* = 38			*n* = 52
Gender			0.74	0.39	
Male	7 (50.0%)	14 (36.8%)			21 (40.4%)
Female	7 (50.0%)	24 (63.2%)			31 (59.6%)
Symptoms					
Foreign body sensation	5 (42.9%)	22 (57.5%)	0.93	0.34	28 (53.8%)
Cervical nodes	8 (57.1%)	23 (60.5%)	0.05	0.83	31 (59.6%)
Local invasion	6 (42.9%)	3 (7.9%)	6.47	0.01	9 (17.3%)
Primary tumor					
Average size (cm, mean ± SD)	1.91 ± 0.65	2.33 ± 1.04	1.39	0.17	2.22 ± 0.96
Unilateral	10 (71.4%)	31 (81.6%)	0.17	0.68	41 (78.8%)
Bilateral	4 (28.6%)	7 (18.4%)			11 (21.2%)
Cervical LN metastases			4.84	0.09	
Ipsilateral	4 (28.6%)	10 (26.3%)			14 (26.9%)
Bilateral	0	7 (18.4%)			7 (13.5%)
Tumor stage			11.45	0.04	
T_1a_	0	4 (10.5%)			4 (7.1%)
T_1b_	5 (35.7%)	9 (23.7%)			14 (26.9%)
T_2_	3 (21.4%)	19 (50.0%)			22 (42.3%)
T_3_	0	2 (5.3%)			2 (3.8%)
T_4a_	5 (35.7%)	3 (7.9%)			8 (15.4%)
T_4b_	1 (7.1%)	1 (2.6%)			2 (3.8%)
N stage			2.45	0.29	
N_0_	10 (71.4%)	18 (47.4%)			28 (53.8%)
N_1a_	1 (7.1%)	5 (13.2%)			6 (11.5%)
N_1b_	3 (21.4%)	15 (39.5%)			18 (34.6%)
M stage			5.65	0.98	
M_0_	12 (85.7%)	38 (100.0%)			50 (96.2%)
M_1_	2 (14.3%)	0			2 (3.8%)
ATA Risk level			4.70	0.09	
Low	8 (57.1%)	17 (44.7%)			25 (48.1%)
Intermediate	1 (7.1%)	13 (34.2%)			14 (26.9%)
High	5 (35.8%)	8 (21.1%)			13 (25.0%)
Operative procedures			10.10	0.01	
Lobectomy	5 (35.7%)	1 (2.6%)			6 (11.5%)
Lobectomy with VI zone dissection	2 (14.3%)	5 (13.2%)			7 (13.5%)
TT with VI zone dissection	7 (50.0%)	32 (84.2%)			39 (75.0%)
Complications					
Transient RLN injury	3 (21.4%)	0	5.1	0.02	3 (5.8%)
Transient hypocalcemia	0	2 (5.3%)	0	0.95	2 (3.8%)
Pulmonary metastasis	2 (14.3%)	8 (21.1%)	0.02	0.88	10 (19.2%)
Recurrence	5 (35.7%)	10 (26.3%)	0.10	0.74	15 (28.8%)

TNM stage and ATA risk level were based on the 8th edition of AJCC thyroid cancer (11). LN, lymph nodes; ATA, American Thyroid Association; TT, total thyroidectomy; RLN, recurrent laryngeal nerve.

Among the 52 cases, 37 patients (9.49 ± 2.63 years old) had no recurrence and 15 patients (8.53 ± 2.56 years old) recurred (Table 3). There was no significant difference in gender between the recurrence group and the non-recurrence group as well as initial symptoms and local invasion (*p* > 0.05). Moreover, no significant difference was present in regard to tumor size and cervical lymph node metastases between the two groups (*p* > 0.05). No statistically significant difference was found in the clinical stage between the recurrence group and the non-recurrence group (*p* > 0.05). Furthermore, there was no significant difference existed in ATA risk levels between the two groups (*p* > 0.05). The 1- and 3-year PFS rates of total thyroidectomy patients were 89.4 and 81.3%, respectively. The proportion of total thyroidectomy in the non-recurrence group (31 cases, 83.8%) was found to be higher than that in the recurrence group (8 cases, 53.3%, *p* ≤ 0.001). There was no statistical difference in the incidence rate of transient RLN injury and hypocalcemia between the two groups (*p* > 0.05). As to comprehensive treatments, we found no concordance between I^131^ radiotherapy or thyroxine tablets and recurrence. In the recurrence group, residual thyroidectomy and cervical lymph node dissection were performed in patients with lobectomy (7 cases, 46.7%), including 5 patients who underwent I^131^ radiotherapy following reoperation, and no one recurred with the combined strategy of thyroxine tablets.

**TABLE 3:** Clinical analysis of the recurrent group and non-recurrent group of DTC in children.

Characteristic	Non-recurrence group	Recurrence group	X^2^(t)	*P*	Total
	*n* = 37	*n* = 15			*n* = 52
Age (years, mean ± SD)	9.49 ± 2.63	8.53 ± 2.56	1.21	0.24	9.21 ± 2.62
Gender			0.37	0.56	
Male	14 (37.8%)	7 (46.7%)			21 (40.4%)
Female	23 (66.7%)	8 (53.3%)			31 (59.6%)
Symptoms					
Foreign body sensation	21 (56.8%)	7 (46.7%)	0.07	0.71	28 (53.8%)
Cervical nodes	15 (40.5%)	7 (46.7%)	0.10	0.75	11 (42.3%)
Local invasion	1 (2.7%)	1 (6.7%)	0.41	0.52	2 (3.8%)
Primary tumor					
Average size (cm, mean ± SD)	2.30 ± 1.07	2.01 ± 0.59	1.00	0.32	2.22 ± 0.96
Unilateral	28 (75.7%)	13 (86.7%)	0.25	0.61	41 (78.8%)
Bilateral	9 (24.3%)	2 (13.3%)			11 (21.2%)
Cervical LN metastases			1.00	0.61	
Ipsilateral	10 (27.0%)	4 (26.7%)			14 (26.9%)
Bilateral	6 (16.2%)	1 (6.7%)			7 (13.5%)
Pulmonary metastasis	5 (13.5%)	5 (33.3%)	1.57	0.21	10 (19.2%)
Tumor stage			2.09	0.84	
T_1a_	3 (8.1%)	1 (6.7%)			4 (7.7%)
T_1b_	10 (27.0%)	4 (26.7%)			14 (26.9%)
T_2_	16 (43.2%)	6 (40.0%)			22 (42.3%)
T_3_	2 (5.4%)	0			2 (3.8%)
T_4a_	5 (13.5%)	3 (20%)			8 (15.4%)
T_4b_	1 (2.7%)	1 (6.7%)			2 (3.8%)
N stage			0.07	0.97	
N_0_	20 (54.1%)	8 (53.3%)			28 (53.8%)
N_1a_	4 (10.8%)	2 (13.3%)			6 (11.5%)
N_1b_	13 (35.1%)	5 (33.3%)			18 (34.6%)
M stage			0.00	1.00	
M_0_	37 (100.0%)	13 (86.7%)			50 (96.2%)
M_1_	0	2 (13.3%)			2 (3.8%)
ATA Risk level			0.85	0.65	
Low	19 (51.4%)	6 (40.0%)			24 (48.0%)
Intermediate	10 (27.0%)	4 (26.7%)			14 (28.0%)
High	8 (21.6%)	5 (33.3%)			13 (25.0%)
Operative procedures			11.75	0.00	
Lobectomy	5 (13.5%)	1 (6.7%)			6 (11.5%)
Lobectomy with VI zone dissection	1 (2.7%)	6 (40.0%)			7 (13.5%)
TT with VI zone dissection	31 (83.8%)	8 (53.3%)			39 (75.0%)
Complications					
Transient RLN injury	1 (2.7%)	2 (13.3%)	0.69	0.41	3 (5.8%)
Transient hypocalcemia	2 (5.4%)	0	0.01	0.90	2 (3.8%)
I^131^ radiotherapy	17 (45.9%)	5 (33.3%)	0.70	0.40	22 (42.3%)
Thyroxine tablets	25 (67.6%)	8 (53.3%)	0.93	0.33	33 (63.5%)

TNM stage and ATA risk level were based on the 8th edition of AJCC thyroid cancer (11). LN, lymph nodes; ATA, American Thyroid Association; TT, total thyroidectomy; RLN, recurrent laryngeal nerve.

Variables correlated with recurrence built a multivariate Cox regression model. Cervical lymph node metastases (both ipsilateral and bilateral metastases, HR 1.12; 95% CI 0.08–15.18 and HR 3.46; 95% CI 0.30–39.77) and young age (HR 1.06; 95% CI 0.28–3.09) were found to be contributing factors for recurrence (Table 4).

**TABLE 4:** Multivariate Cox regression models for predicting recurrence.

Value	*B*-value	SE	Wald	*P*	HR	HR 95% CI
Tumor size	–1.01	0.49	4.24	0.04	0.35	0.14–0.95
Ipsilateral cervical LN metastases	0.11	1.33	0.00	0.93	1.12	0.08–15.18
Bilateral cervical LN metastases	1.24	1.25	0.99	0.32	3.46	0.30–39.77
I^131^ radiotherapy	–0.02	0.83	0.00	0.99	0.99	0.19–5.01
Thyroxine tablets	–1.84	0.93	3.98	0.05	0.16	0.03–0.97
Pre-school group	0.06	0.68	0.01	0.93	1.06	0.28–3.99

LN, lymph nodes.

**FIGURE 1:**
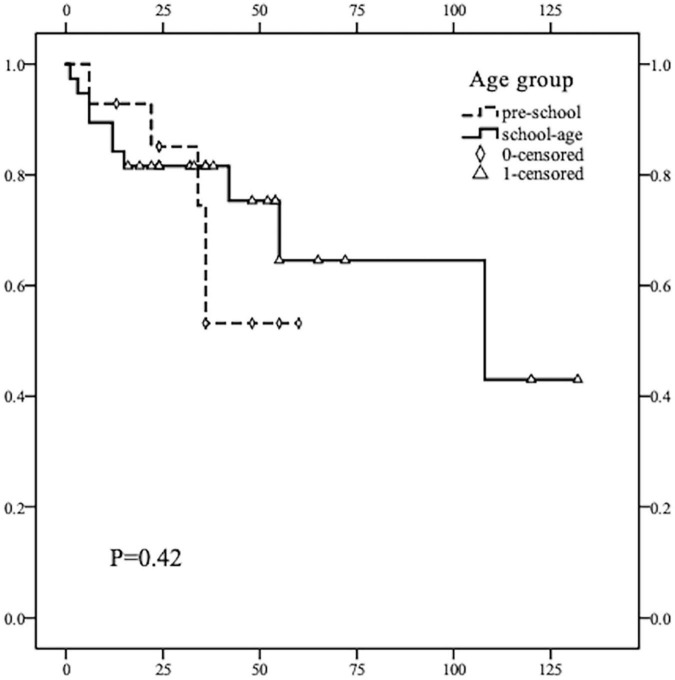
Kaplan–Meier survival curve comparison of PFS rate in children of different age groups (≤ 7 years old and 8–13 years old). The recurrence rate of thyroid cancer in children of the two groups was not statistically significant (*p* = 0.42, *p* > 0.05). The average and median times of recurrence DTC in the pre-school children were earlier than that in the school-age children.

All children were followed up for a mean of 37.9 months after surgery, and all cases survived up to the follow-up time point, with a 5-year PFS rate of 61.86% for all cases. The 5-year PFS rate was 52.9% in the pre-school group and 64.0% in the school-age group. No deaths were observed in the two groups during the follow-up period. Kaplan–Meier curve shows no statistically significant difference in PFS between the two age groups (*p* > 0.05) (Figure 1).

**FIGURE 2:**
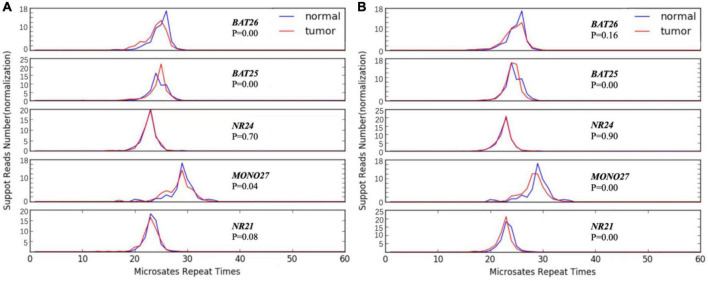
Polymerase chain reaction analysis of microsatellite markers. **(A)** The blue curve represents normal tissue, while the red curve represents tumor tissue. In case 1, there was microsatellite instability in *BAT26, BAT25*, and *MONO27* sites between two control tissues (*p* < 0.05), which indicated high microsatellite instability (MSI-H). **(B)** In case 2, there was microsatellite instability in *BAT25, MONO27*, and *NR21* sites between two control tissues (*p* < 0.05), which indicated high microsatellite instability (MSI-H).

Notably, two pre-school children recurred after the first surgery with cervical lymph node metastases and tracheal invasion, making salvage surgery encounter more difficulties; both cases developed postoperative pulmonary metastasis after complete resection of residual tumor tissue. Microsatellite instability has been used as an important molecular marker for the prognosis of some refractory tumors and for predicting the therapeutic effect of immunotherapy (12). Previous research has reported that patients with thyroid cancer may benefit from immunotherapy (13). To further explore the characteristics and treatment strategies of these two patients, single-nucleotide marker sites (*BAT25, BAT26, MONO-27, NR21, NR24*) were amplified by MSI-PCR. Not surprisingly, the examined single-nucleotide marker sites of both samples exceeded 30% (more than 2 of the 5 sites) and were defined as high microsatellite instability (MSI-H) (Figure 2).

## Discussion

The incidence of DTC in children and adolescents has continuously risen in recent years, which largely attributes to the improvements in diagnostic methods. Previous studies have pointed out that DTC in children and adolescents is different from that of adults in clinical manifestations, recurrence rate, pulmonary metastasis, and prognosis (14, 15). An analysis conducted by Al-Qahtani et al. in children and adolescents with DTC under 18 years old has indicated that young age was dramatically related to the recurrence *in situ* (14). Domestic studies have compared specificity and difference in clinical characteristics of DTC between preadolescent children and adolescents; the incidence of distant metastases was reported to be higher in those of young age (16). This study first provides insights into the clinical features in DTC children younger than or equal to 13 years of age. Clinical characteristics of the pre-school group and the school-age group were compared and analyzed; prognosis analysis between recurrence and non-recurrence groups was conducted as well. We hope this study could provide a more comprehensive acknowledgment for the risk factors and distribution characteristics in children with DTC.

In this study, the cervical node was the most common initial clinical manifestation in pre-school children, whereas in the school-age group, the initial symptom of foreign body sensation and cervical lymph node was most common. In light of the non-typical symptoms and anatomical location, thyroid cancer in children is often diagnosed in an advanced stage, especially in pre-school age children without well-managed speech expression ability. The proportion of local invasion was higher in the pre-school group than that in the school-age group, demonstrating that compared to the school-age group, tumors in the pre-school group were more subtle and aggressive (17). In this study, a significant difference was observed in the T stage between the two age groups, with a higher proportion of T_1–2_ in the school-age group and a higher proportion of T_4_ in the pre-school group. Similarly, there was no statistically significant difference regarding ATA risk level between the two groups in this study. However, in terms of percentage, the proportion of high-risk cases appeared to be higher in the pre-school group than that in the school-age group. Therefore, young age serves as a critical risk factor in the T stage and ATA risk level of DTC in children, which has some similarities to another large retrospective cohort study by Lebbink et al. (5). Positive physical examination and ultrasonic examination should be attached of importance for the early diagnosis and treatment of DTC in pre-school children. Notably, it is acknowledged that there is overdiagnosis in children with DTC due to the overdiagnosis with ultrasonography screening, leading to lifelong side effects, medical care, and even psychological problems (18). For clinical implications, children with potential specific risk factors (such as adolescence in girls, Hashimoto thyroiditis, and family history) are feasible for active screening (19).

In terms of surgery procedures, the total thyroidectomy proportion was 50.0 and 84.2% in the pre-school group and school-age group, respectively, which may be relevant to conservative surgical methods under the aspiration of pre-school age children’s parents. As to the recurrence risks, the proportion of bilateral total thyroidectomy in the non-recurrence group was found to be significantly higher than that in the recurrence group by univariate factor analysis, while cervical lymph node metastases and young age were found to be risk factors of recurrence by the multivariate regression model. However, the surgical procedure analysis has certain limitations of the small cohort; also, this study involved children in a broad period, during which much has changed in terms of surgical instruments, equipment, and multimodal regimens over the years. It is necessary to conduct further studies with higher patient volumes and multicenter studies to support the optimum strategy. As reported, no difference was found between unilateral thyroidectomy and total thyroidectomy with respect to disease-free survival (DSS), and both procedures had a good prognosis in children (20).

There is still controversy concerning the therapeutic approach in children with DTC, regarding the resection range and the necessity of postoperative I^131^ radiotherapy (21). The 2015 ATA guidelines recommended less use of I^131^ radiotherapy for low-risk DTC patients because the impact of I^131^ radiotherapy on children remains unclear, which may affect the quality of life and carry an economic burden (22). It has been reported that a similar low recurrence rate between total thyroidectomy and total thyroidectomy is associated with preventive central neck dissection in adults (23). Besides, NGO et al. pointed out that lymph node metastases in the central region of papillary thyroid cancer (PTC) children were as high as 83.3% (24). A cohort study of 102 children PTC cases has suggested that for patients with unifocal T_1a_ without clinically evident nodal disease, a unilateral thyroidectomy with VI zone dissection should be considered (25). Generally speaking, in patients with tumor size ≥ 4 cm and/or multifocal neoplasia, and/or local invasion, and/or lymph node metastases, and/or distant metastases, total thyroidectomy followed by I^131^ radiotherapy is suggested (26). For our statistics, surgical complications were more frequent in the pre-school group than that in the school-age group, which may be related to recurrent laryngeal nerve invasion and complex anatomical structure in younger children. The overall incidence rate of recurrence and pulmonary metastasis in this study was 28.8 and 19.2%, which echoed to the proportion reported in previous studies (27).

Compared to the non-recurrence group, those in the recurrence group were slightly younger (8.53 ± 2.56 years old), indicating that younger DTC patients should attach great importance to regular follow-ups and examinations. In this study, no statistical difference in gender was noted between non-recurrent and recurrent groups, which was consistent with the work of Demidchik et al. (28). Moreover, no statistical difference was present in the clinical stage and ATA risk level between recurrent and non-recurrent groups. This study stumbled upon a high proportion of T_1a_, T_1b_, and T_2_ in the recurrent group (73.4%), which indicated that more recurrent cases had a low T stage at initial diagnosis. Also, the ATA risk level is insufficient in assessing the risk of DTC recurrence in children adequately. It is reported that the overall cumulative survival rate for children with thyroid cancer was high up to 97–99% (20), which appeared to be 100% in our study after surgical resection and I^131^ radiotherapy with thyroxine tablets. Most of the previous studies focused on exploring the survival rate for children with thyroid cancer but pay less attention to the prognosis factors on account of the very low mortality.

The curative effects of traditional treatment in metastatic thyroid cancers are limited. Immunotherapy is expected to become a new treatment modality to improve the prognosis of these patients. MSI is important in selecting patients who may benefit from immune checkpoint inhibitors. A publication by Genutis et al. described a total of 485 thyroid cancer patients screened for MSI deficiency, which found that all MSI-H cases were follicular thyroid carcinoma (FTC) patients (28). The insights gained from the result of MSI in two refractory cases may highlight the importance of MSI examination for immunotherapy in recurrent and aggressive DTC patients. Further clinical data and trials are expected to support the application of immunotherapy in thyroid cancer. There was a potential bias of age, tumor stage, body mass index, and economic situations. Therefore, larger clinical trials should be designed to explore the feasibility and efficacy of therapeutic regimens.

## Conclusion

In conclusion, the proportion of local invasion, tumor stage, and recurrent laryngeal nerve injury in the pre-school age group was higher than that of the school-age group. Young age may be presumed to be a risk factor in children with DTC. Nearly half of the recurrent cases were assessed as low T stage and low-risk at initial diagnosis; hence, strategies in evaluating the risks of children with DTC locoregional relapse should be further investigated. To raise awareness of the risk of DTC in young children, further studies and appropriate treatment measures should be designed.

## Data availability statement

The raw data supporting the conclusions of this article will be made available by the authors, without undue reservation.

## Ethics statement

The studies involving human participants were reviewed and approved by the Ethics Committee of Xinhua Hospital Affiliated with the Shanghai Jiao Tong University of Medicine (No. XHEC-WJW-2022-272, February 28, 2022). Written informed consent to participate in this study was provided by the participants’ legal guardian/next of kin.

## Author contributions

CC and JL contributed to the conception and design of the study and wrote the first draft of the manuscript. LH performed the statistical analysis. JX and JY participated in drafting the manuscript or revising for intellectual content. YZ and YW contributed to the manuscript revision. QZ supervised the writing and editing. JX modified and improved the English. All authors contributed to manuscript revision, read, and approved the submitted version.
